# The expression pattern and role of circulating CXCR5^+^*γδ* T cells in children with newly diagnosed immune thrombocytopenia

**DOI:** 10.3389/fped.2025.1646877

**Published:** 2025-08-11

**Authors:** Jian-Yong Wang, Yi Xin, Xiao-Li Wang, Lin-Lin Li, Ai-Min Li, Xiao-Lu Zhang

**Affiliations:** ^1^ Department of Pediatrics, The Affiliated Yantai Yuhuangding Hospital of Qingdao University, Yantai, China; ^2^ Department of Clinical Laboratory, The Affiliated Yantai Yuhuangding Hospital of Qingdao University, Yantai, China

**Keywords:** immune thrombocytopenia, newly diagnosed, children, CXCR5^+^*γδ* T cells, Tfh cells

## Abstract

**Introduction:**

Immune thrombocytopenia (ITP) is the most common bleeding disorder in children. Tfh cells play a crucial role in the pathogenesis of ITP by promoting the production of anti-platelet autoantibodies. Recent studies have shown that CXCR5^+^*γδ* T cells not only possess “Tfh-like” cell functions but also can induce Tfh cell differentiation. However, it remains unknown whether CXCR5^+^*γδ* T cells are involved in the pathogenesis of ITP. This study aims to investigate the role of CXCR5^+^*γδ* T cells in children with newly diagnosed ITP (nITP).

**Methods:**

A total of 96 children with nITP and 48 healthy children were enrolled in this study. FCM was used to compare the frequencies of circulating CXCR5^+^*γδ* T cells and circulating Tfh cells, as well as the levels of ICOS and CD40l on circulating CXCR5^+^*γδ* T cells in both groups. The correlation between circulating CXCR5^+^*γδ* T cells and platelets as well as circulating Tfh cells were further analyzed.

**Results:**

Compared with healthy controls, the frequency of circulating CXCR5^+^*γδ* T cells was higher in children with nITP, and it was negatively correlated with platelet count. The levels of ICOS and CD40l on circulating CXCR5^+^*γδ* T cells in children with nITP were also higher. Children with nITP had a higher frequency of circulating Tfh cells, which was positively correlated with circulating CXCR5^+^*γδ* T cells.

**Conclusions:**

The excessive activation and proliferation of CXCR5^+^*γδ* T cells may contribute to the pathogenesis of nITP in children. Therefore, it can be used as a target for the immunotherapy of pediatric ITP.

## 1 Introduction

Immune thrombocytopenia (ITP) is the most common hemorrhagic disorder in childhood, characterized by a reduced peripheral blood platelet count (less than 100 × 10^9^/L) due to increased platelet clearance and impaired platelet production (1). The typical clinical presentation includes spontaneous skin and mucous membranes bleeding, with occasional visceral hemorrhage. Based on the duration of condition, ITP is classified as newly diagnosed ITP (nITP) (within 3 months from diagnosis), persistent ITP (between 3 and 12 months) or chronic ITP (more than 12months) (2). The prognosis of pediatric ITP is usually benign, with the majority of cases achieving complete recovery within 12 months of diagnosis (3). Unfortunately, approximately 20% of nITP children eventually progress to chronic ITP, resulting in a significant decline in their quality of life (4). A deeper understanding of its pathogenesis will help to improve awareness and treatment effectiveness of ITP in children. Therefore, it is particularly important to further investigate the pathogenesis of childhood ITP.

As an autoimmune disease, ITP has a complex pathogenesis which involves both innate and adaptive immune system disorders, including humoral and cellular immunity (5). Platelets and their precursors become targets of the dysregulated immune system in ITP, leading to increased platelet destruction and reduced megakaryogenesis and thrombopoiesis (5, 6). Among them, the anti-platelet autoantibodies produced by autoreactive B cells against platelet membrane glycoprotein (GP) play the most important role in this process (7). These autoantibodies are mainly of the specific immunoglobulin G (IgG) type, but in a few cases also other isotypes (IgA and IgM) (5). Since the production of anti-platelet antibodies involves affinity maturation and Ig class switching, B cells require the help of T cells to generate these pathogenic antibodies, implicating the importance of these T cells in the pathogenesis of ITP (8). T follicular helper (Tfh) cells, a unique subset of CD4 ^+^ T cells, specialize in providing crucial assistance for the differentiation of B cells (9). The characteristic hallmark of Tfh cells is the expression of the surface molecule C-X-C motif chemokine receptor 5 (CXCR5), which enables them to migrate into follicles in secondary lymphoid tissues (10). Tfh cells support germinal center (GC) responses in lymphoid follicles to facilitate antibody production (11). However, abnormal proliferation and function of Tfh cells can lead to the production of autoantibodies. As a result, these cells are involved in the onset and progression of numerous autoimmune diseases (12). Studies have confirmed that Tfh cells are abnormally expanded during ITP and play a crucial role in the pathogenesis of ITP by promoting the production of anti-platelet autoantibodies by autoreactive B cells (6–8).

Besides Tfh cells, gamma delta (*γδ*) T cells also play an important role in T cell-dependent antibody production. *γδ* T cells represent a minor yet distinctive subset of T cells in peripheral blood (PB), distinguished by the expression of T cell receptor (TCR) heterodimers consisting of the TCR *γ* and *δ* chains (13). These cells functionally bridge the innate and adaptive immunity, participating in diverse immune response and regulatory processes, thereby fulfilling a distinctive and protective role in immune surveillance (14). Importantly, *γδ* T cells also contribute to antibody production by initiating and maintaining the GC reaction (15). However, *γδ* T cells are more helpful in promoting the production of antibodies against “self” by B cells (15, 16). Consistent with this, *γδ* T cells play an important role in the pathogenesis of antibody-mediated autoimmune diseases (17). A previous study has also confirmed the involvement of *γδ* T cells in the pathogenetic mechanism of some children with ITP (18). Significantly, *γδ* T cells regulate humoral immune response primarily through CXCR5^+^*γδ* T cell subset (19, 20). CXCR5^+^*γδ* T cells not only possess “Tfh-like” cells functions but also induce Tfh cell differentiation to help B cells produce antibodies (19–21). To date, there has been no research on CXCR5^+^*γδ* T cells in ITP.

Based on the above studies, we speculate that CXCR5^+^*γδ* T cells may be involved in the pathogenesis of children with nITP. To this end, we assessed the expression levels of circulating CXCR5^+^*γδ* T cells and their cell surface functional molecules inducible co-stimulators (ICOS) and CD40 ligand (CD40l) in children with nITP, and analyzed their correlation with platelet count and circulating Tfh cells.

## 2 Materials and methods

### 2.1 Ethics approval

This study was approved by the Ethics Committee of Yantai Yuhuangding Hospital (Ethical approval No.: 2021-019 and approved on January 26, 2021). The written informed consent forms were signed by the parents or legal guardians of all participating children.

### 2.2 Study participants

A cohort of 96 children with nITP at Yantai Yuhuangding Hospital were enrolled in the study from March 2021 to June 2024. All of our pediatric ITP patients were diagnosed according to the American Society of Hematology 2019 guidelines for ITP (22). Cases with the following conditions were excluded: (1) who had been treated with glucocorticoid or intravenous immunoglobulin before specimen collection; (2) who have congenital diseases, autoimmune diseases, tumors, and other underlying diseases. A total of 48 healthy children were recruited as healthy controls at the same period. The clinical and laboratory characteristics of the 96 children with nITP and the 48 healthy controls were presented in Table 1. There was no significant difference in the distribution of age, sex, and white blood cell count (WBC) between the two groups.

**Table 1:** Baseline characteristics of children with nITP and healthy controls.

Variables	Healthy controls (*n* = 48)	nITP (*n* = 96)	*P*-value
Age (months)	30.00 (14.50–53.50)	36.50 (14.25–63.00)	0.725
Gender (male/female)	25 (52.08%)/23 (47.92%)	54 (56.25%)/42 (43.75%)	0.636
WBC (×10^9^/L)	7.02 ± 2.13	7.76 ± 3.29	0.107
PLT (×10^9^/L)	209.58 ± 53.14	19.38 ± 11.23	<0.001

Fasting venous blood samples were gathered from children with nITP before treatment and healthy pediatric controls. Whole PB samples were stored at 4˚C for flow cytometric analysis.

### 2.3 Flow cytometry (FCM)

Whole PB samples (100 μl) were incubated with specific antibodies at room temperature in the dark for 30 min. Subsequently, red blood cells were lysed using lysis solution (BD Biosciences, Cat.349202) for 10 min, and the samples were washed twice with phosphate-buffered saline (PBS). The expression of cell surface markers was analyzed by FCM using a BD FACSLyric flow cytometer (BD Biosciences). The following antibodies obtained from BioLegend (San Diego, CA, USA) were used: PerCP anti-human CD45 (Cat.304026), APC/Cy7 anti-human CD3 (Cat.300426), APC anti-human TCR*γ*/*δ* (Cat.331212), FITC anti-human CD4 (Cat.344604), PE anti-human CXCR5 (Cat.356904), PE/Cy7 anti-human ICOS (Cat.313520) and BV421^TM^ anti-human CD40l (Cat.310824).

### 2.4 Statistical analysis

Statistical analyses were performed using SPSS software (version 23.0). Normal distribution data were presented as mean ± SD and independent t-test was used to compare these data. Skewed distribution data were presented as median (quartile) and compared by the Mann–Whitney test. Categorical data were presented as number (%) and compared using Chi-squared tests. The strength and direction of the linear relationship between two continuous variables were analyzed by the Pearson's correlation coefficient. A *p-*value <0.05 was considered to be statistically significant.

## 3 Results

### 3.1 Increased frequency of circulating CXCR5^+^*γδ* T cells in children with nITP

**Figure 1:**
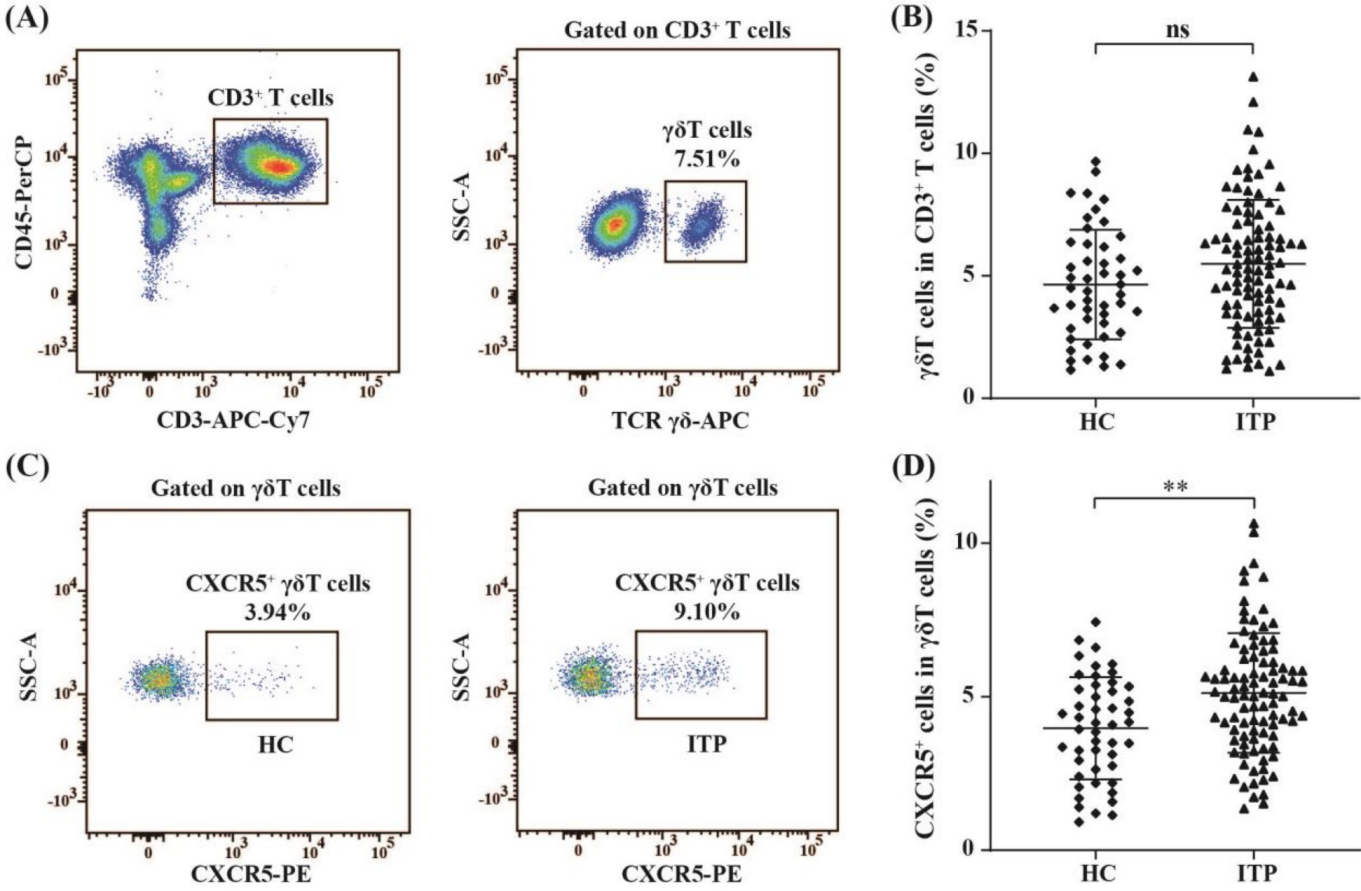
The percentages of circulating *γδ* T cells and CXCR5^+^*γδ* T cells in PB of children with nITP and healthy controls. **(A)** Flow cytometric gating strategy: CD3 ^+^ T lymphocytes were first gated and then *γδ* T cells were gated in CD3 ^+^ T lymphocytes. **(B)** Percentages of circulating *γδ* T cells in the ITP and control groups. **(C)** Representative plots of circulating CXCR5^+^*γδ* T cells from each group. **(D)** Percentages of circulating CXCR5^+^*γδ* T cells in the ITP and control groups. Each data point represents one individual. Results are expressed as the mean ± SD. **p < 0.01, ns = not significantly different.

To investigate the role of CXCR5^+^*γδ* T cells in the pathogenesis of nITP in children, we first analyzed the proportion of *γδ* T cells in PB CD3 ^+^ T lymphocytes of pediatric patients with nITP (n = 96) and healthy controls (*n* = 48) by FCM. By gating the CD3 ^+^ T cell population, the frequency of *γδ* T cells in PB was determined (Figure 1A). As shown in Figure 1B, there was no significant difference in the percentage of circulating *γδ* T cells between children with nITP and healthy controls (5.49 ± 2.61% vs. 4.64 ± 2.23%, *p* = 0.057). Subsequently, we further analyzed the expression of CXCR5 on PB *γδ* T cells. Our findings revealed that the frequency of CXCR5^+^ cells among PB *γδ* T cells was significantly higher in children with nITP compared to healthy controls (5.12 ± 1.95% vs. 3.97 ± 1.67%, *p* = 0.001, Figures 1C,D).

### 3.2 Negative correlation between the circulating CXCR5^+^*γδ* T cells and platelet count in children with nITP

**Figure 2:**
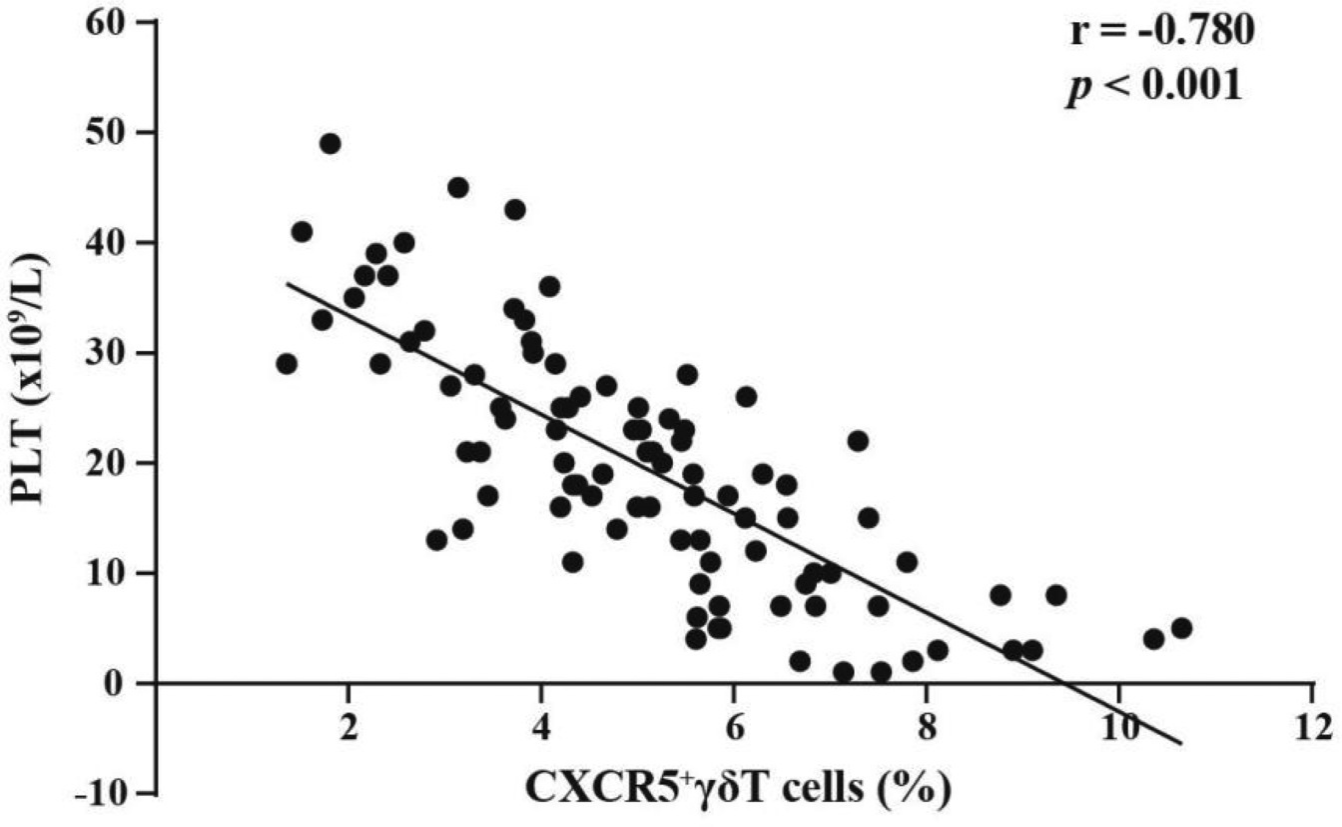
Correlation analysis between the percentages of circulating CXCR5^+^*γδ* T cells and platelet count in children with nITP.

The relationship between the percentages of circulating CXCR5^+^*γδ* T cells and platelet count in children with nITP were assessed. We found a significant negative correlation between the percentages of circulating CXCR5^+^*γδ* T cells and platelet count (r = − 0.780, *p* < 0.001, *n* = 96), as shown in Figure 2.

### 3.3 Increased expressions of costimulatory molecules ICOS and Cd40l on CXCR5^+^*γδ* T cells from children with nITP

**Figure 3:**
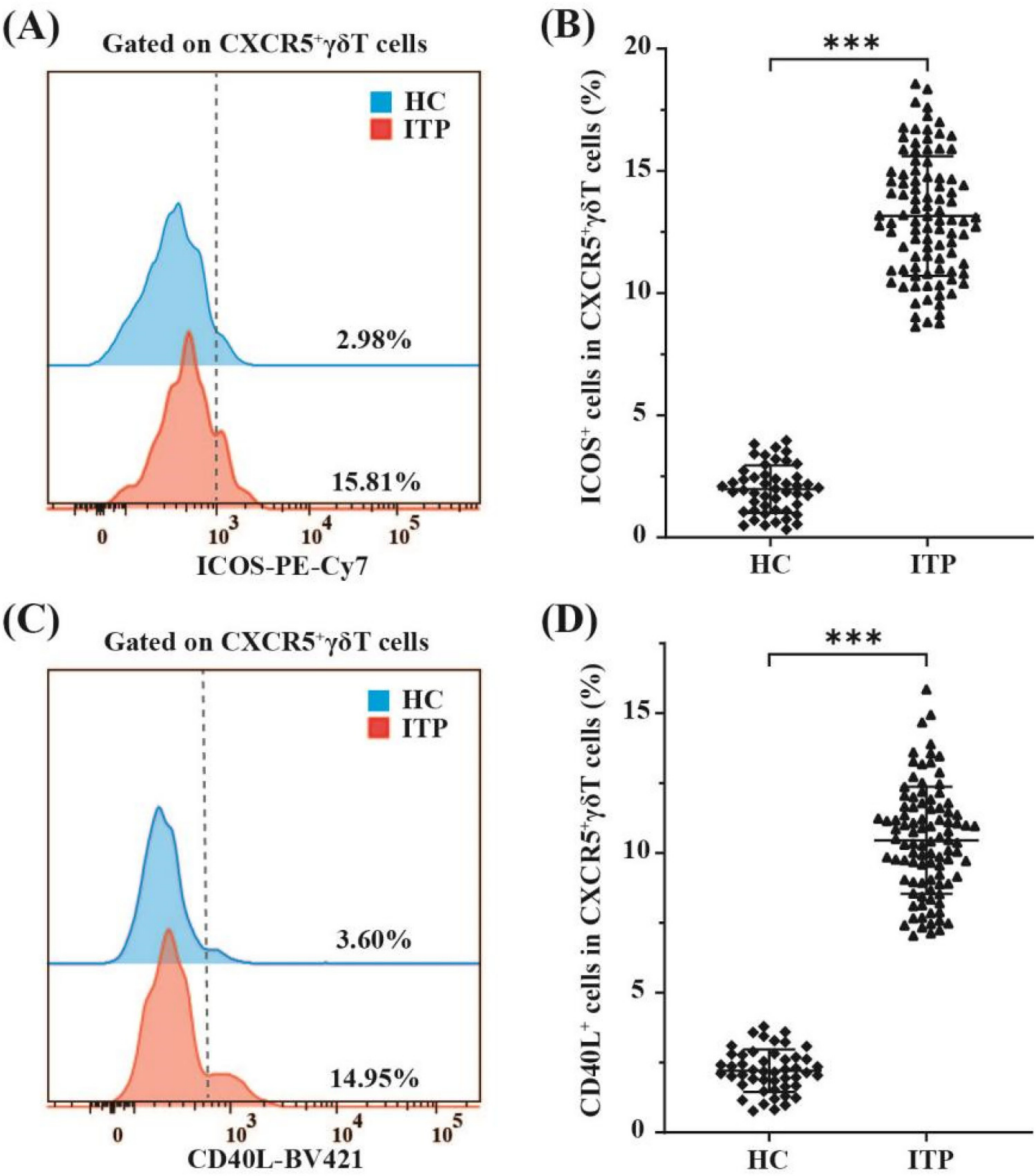
FCM analysis of ICOS and CD40l expression on circulating CXCR5^+^*γδ* T cells in children with nITP and healthy controls. **(A)** Representative histograms of ICOS expression on CXCR5^+^*γδ* T cells from PB of children with nITP (red) and healthy controls(blue). **(B)** Expression levels of ICOS in circulating CXCR5^+^*γδ* T cells between the ITP and control groups. **(C)** Representative histograms of CD40l expression on CXCR5^+^*γδ* T cells from PB of children with nITP (red) and healthy controls(blue). **(D)** Expression levels of CD40l in circulating CXCR5^+^*γδ* T cells between the ITP and control groups. Each point on the dot plot represents an individual subject. Data are shown as the mean ± SD. ***p < 0.001.

To elucidate the functional characteristics of circulating CXCR5^+^*γδ* T cells, we next detected the expression levels of the costimulatory molecules ICOS and CD40l on these cells in ITP pediatric patients (*n* = 96) and healthy controls (*n* = 48) by FCM. The proportions of ICOS (13.16 ± 2.45% vs. 1.98 ± 0.97%, *p* < 0.001, Figures 3A,B) and CD40l (10.45 ± 1.92% vs. 2.21 ± 0.76%, *p* < 0.001, Figures 3C,D) on circulating CXCR5^+^*γδ* T cells in children with nITP were significantly higher than those in healthy controls. These findings suggest that CXCR5^+^*γδ* T cells may be overactivated in children with nITP.

### 3.4 Positive correlation between circulating CXCR5^+^*γδ* T-cells and circulating Tfh cells in children with nITP

**Figure 4:**
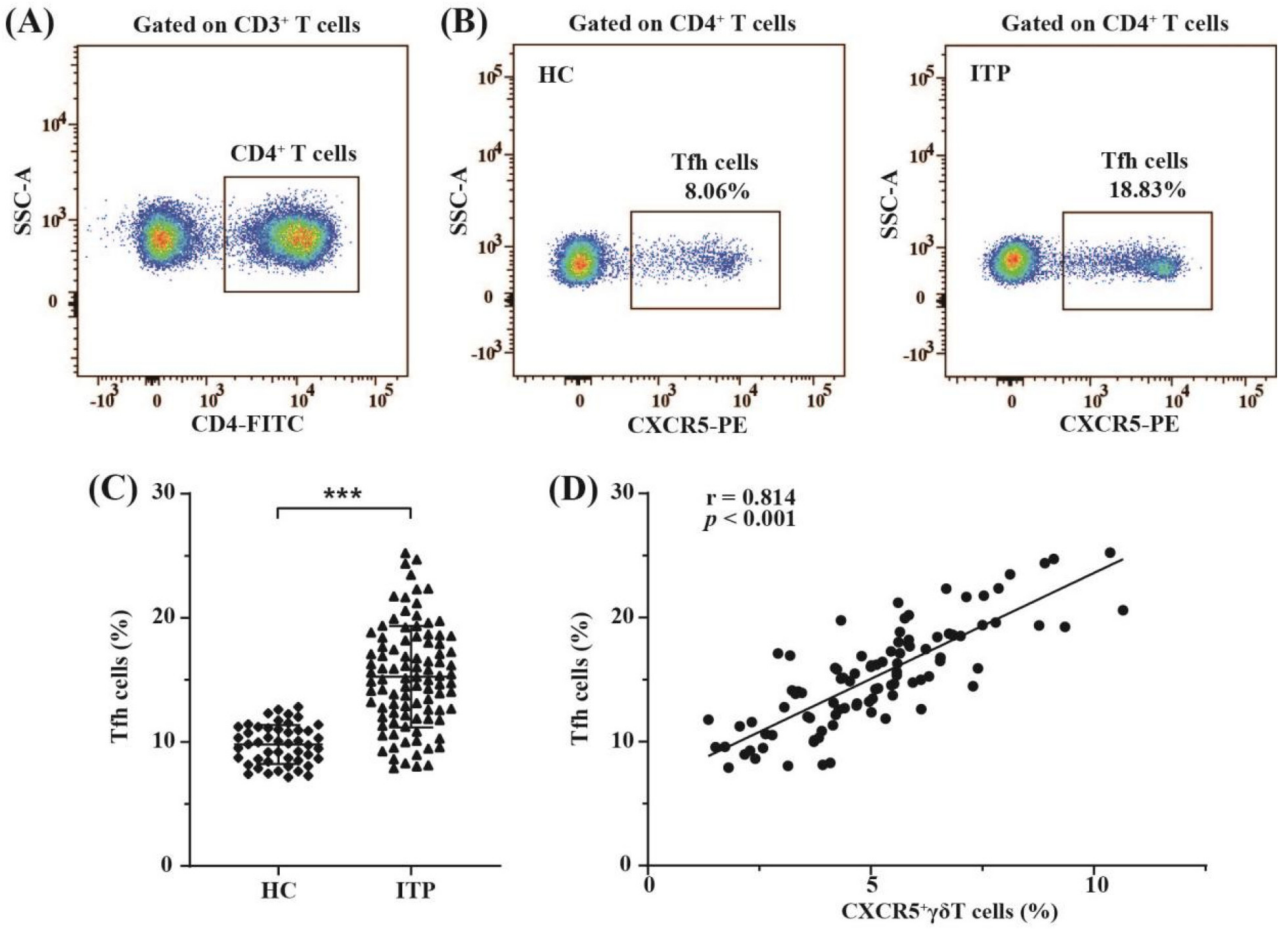
The distribution of circulating Tfh cells and their correlation with circulating CXCR5^+^*γδ* T cells in children with nITP. **(A)** CD3 ^+^ CD4 ^+^ T cells were gated. **(B)** Representative FCM plots of circulating Tfh cells (CD4 ^+^ CXCR5 ^+^ T cells) in children with nITP and healthy controls. **(C)** Percentages of circulating Tfh cells in each group. Each dot represents one individual. Results are expressed as mean ± SD. ***p < 0.001. **(D)** Correlation analysis between the percentages of circulating Tfh cells and the percentages of circulating CXCR5^+^*γδ* T cells in children with nITP.

Recent study has shown that CXCR5^+^*γδ* T cells can induce Tfh cell formation (19). Therefore, the present study assessed expression levels of circulating Tfh cells in children with nITP and compared their expressions to the healthy controls. CD4 ^+^ CXCR5 ^+^ T cells in PB were defined as circulating Tfh cells (9). In order to identify circulating Tfh cells, CD3 ^+^ CD4 ^+^ T cell population were gated on (Figure 4A). Subsequently, the percentages of circulating Tfh cells were determined using FCM (Figure 4B). As indicated in Figure 4C, the proportion of circulating Tfh cells in children with nITP was significantly higher compared to healthy controls (15.25 ± 4.09% vs. 9.79 ± 1.57%, *p* < 0.001). Next, we explored whether an elevated proportion of Tfh cells was linked to an increased proportion of CXCR5^+^*γδ* T cells in PB of children with nITP. Correlation analysis revealed the percentages of circulating Tfh cells were positively correlated with the percentages of circulating CXCR5^+^*γδ* T cells in children with nITP (r = 0.814, *p* < 0.001, *n* = 96, Figure 4D).

## 4 Discussion

Childhood ITP is an acquired autoimmune bleeding disorder. Although its pathophysiology is incompletely understood, immune-mediated increased destruction and decreased production of platelets are recognized mechanisms (5–7). Anti-platelet autoantibodies targeting platelet GPs or GP complexes are considered to be the main cause of thrombocytopenia in its pathogenesis (6, 7). The generation of these specific autoantibodies depends on the help of T cells (8). It is well known that *γδ* T cells exert a strong influence on T cell-dependent antibody production (15, 23). CXCR5^+^*γδ* T cells represent the most crucial subset of *γδ* T cells regulating antibody production (19, 20). Due to *γδ* T cells tendency to promote the production of autoantibodies, they play a distinctive and significant role in many autoimmune diseases (15–17). However, little is known about the role of *γδ* T cells in childhood ITP, especially CXCR5^+^*γδ* T cells. In the present study, we for the first time investigated the role of circulating CXCR5^+^*γδ* T cells in children with nITP. To this end, the levels of *γδ* T cells in the PB of children with nITP were initially evaluated. We noticed that, although higher frequency of circulating *γδ* T cells in nITP children compared to healthy controls, there was no statistically significant difference between the two groups, consistent with previous research findings (18). Subsequently, our further investigation revealed that the percentage of circulating CXCR5^+^*γδ* T cells increased significantly in children with nITP and was closely negatively correlated with platelet count. These findings suggest that CXCR5^+^*γδ* T cells may contribute to the immunopathogenesis of nITP in children.

Previous studies have shown that CXCR5^+^*γδ* T cells have “Tfh-like” cells function, also known as *γδ*Tfh cells (20, 21). The function of Tfh cells is specifically to help B cells produce antibodies, which depends on the expression of functional molecules of Tfh cells, including the CXCR5, co-stimulatory molecules ICOS and CD40l, among others (9, 10). CXCR5 is essential for Tfh cells migration to T-B cell junctions in lymphoid follicles. ICOS, by binding to ICOS ligands (ICOSL) on the surface of B cells, can enable Tfh cells to more precisely localize at B cell follicles and engage in cell-cell contact with B cells (24, 25). CD40l interacts with CD40 on the surface of B cells to provide the most important co-stimulatory signal for B cell proliferation and differentiation, which is crucial for the GC response (25, 26). These molecules synergistically promote the production of antibodies, and a lack of any one of them leads to obstacles in antibody generation. Studies have confirmed that CXCR5^+^*γδ* T cells also help B cells produce antibodies through the action of these functional molecules (15, 20). In patients with ITP, Tfh cells exhibit excessive proliferation and promote the production of anti-platelet autoantibodies through functional molecules such as ICOS and CD40l(5–7). Our research revealed that circulating CXCR5^+^*γδ* T cells were also increased in children with nITP, accompanied by elevated expression of their functional molecules ICOS and CD40l. Accordingly, we speculate that similar to Tfh cells, CXCR5^+^*γδ* T cells may facilitate the generation of anti-platelet autoantibodies through these functional molecules, thereby contributing to the pathogenesis of nITP in children.

CXCR5^+^*γδ* T cells can also regulate antibody production by inducing Tfh cell differentiation (19). Although B-cell lymphoma 6 (BCL6), as a specific transcription factor of Tfh cells, plays a central role in their differentiation, it does not participate in regulating the initiation of Tfh cell program (27). During the differentiation process of Tfh cells, the expression of CXCR5 precedes that of Bcl6 (28). The transcription factor achaete-scute homologue 2 (Ascl2) is particularly important for the initiation of Tfh cell differentiation and the induction of CXCR5 (29, 30). As a downstream target of the Wnt signaling pathway, Ascl2 can be induced by Wnt ligands (31). Recent study has confirmed that CXCR5^+^*γδ* T cells can induce the expression of Ascl2 in naive CD4 ^+^ T cells by releasing Wnt ligands, which in turn initiates Tfh cell program and promotes CXCR5 expression (19). Our research found that the frequency of circulating Tfh cells was also significantly increased in nITP children, which was consistent with previous study (32). Furthermore, the level of circulating Tfh cells was closely positively correlated with that of circulating CXCR5^+^*γδ* T cells in these children. These results indicate that the abnormal expansion of CXCR5^+^*γδ* T cells may induce the generation of more Tfh cells in children with nITP. Importantly, Tfh cells have been proven to play a crucial role in the pathogenesis of ITP by promoting the production of anti-platelet autoantibodies (6–8). Therefore, we speculate that CXCR5^+^*γδ* T cells may also be involved in the pathogenesis of nITP in children by inducing the generation of Tfh cells.

In conclusion, our study suggests that CXCR5^+^*γδ* T cells exhibit excessive activation and proliferation in children with nITP, and they may participate in the pathogenesis through functioning as “Tfh-like” cells and inducing Tfh cell differentiation. The latest research has confirmed that the expansion of Tfh cells correlates with the severity of ITP (33), which also explains why circulating CXCR5^+^*γδ* T cells are closely related to the degree of thrombocytopenia in children with nITP. Therefore, it can be inferred that CXCR5^+^*γδ* T cells play a pivotal role in pediatric nITP. However, there are several limitations in the present study. First, this study was conducted in a single center. Although the selection bias was reduced to some extent by increasing the sample size, it is still necessary to verify the general applicability of the research conclusions in multi-center studies. Second, no longitudinal peripheral blood samples were obtained to assess the levels of circulating CXCR5^+^*γδ* T cells during the disease recovery period, making it impossible to dynamically observe the changes of CXCR5^+^*γδ* T cells in the disease course of children with nITP.Second, no longitudinal peripheral blood samples were obtained to assess the levels of circulating CXCR5^+^*γδ* T cells during the disease recovery period, making it impossible to dynamically observe the expression changes of circulating CXCR5^+^*γδ* T cells in the disease course of nITP in children. Third, the specific mechanism of CXCR5^+^*γδ* T cells in children with ITP has not been thoroughly investigated. Further studies are needed in the future to clarify its detailed underlying mechanism.

## 5 Conclusions

Abnormally activated and proliferated CXCR5^+^*γδ* T cells may be involved in the pathogenesis of nITP in children. Therefore, it can be used as a target for the immunotherapy of pediatric ITP, thereby providing new ideas for the clinical treatment of pediatric ITP.

## Data Availability

The original contributions presented in the study are included in the article/Supplementary Material, further inquiries can be directed to the corresponding authors.
